# Supplementary figures and images for: Lack of apoptosis leads to cellular senescence and tumorigenesis in Drosophila epithelial cells
Source: Cell Death Discov. 2023 Aug 2;9:281. doi: 10.1038/s41420-023-01583-y (PMC10397273; doi:10.1038/s41420-023-01583-y)

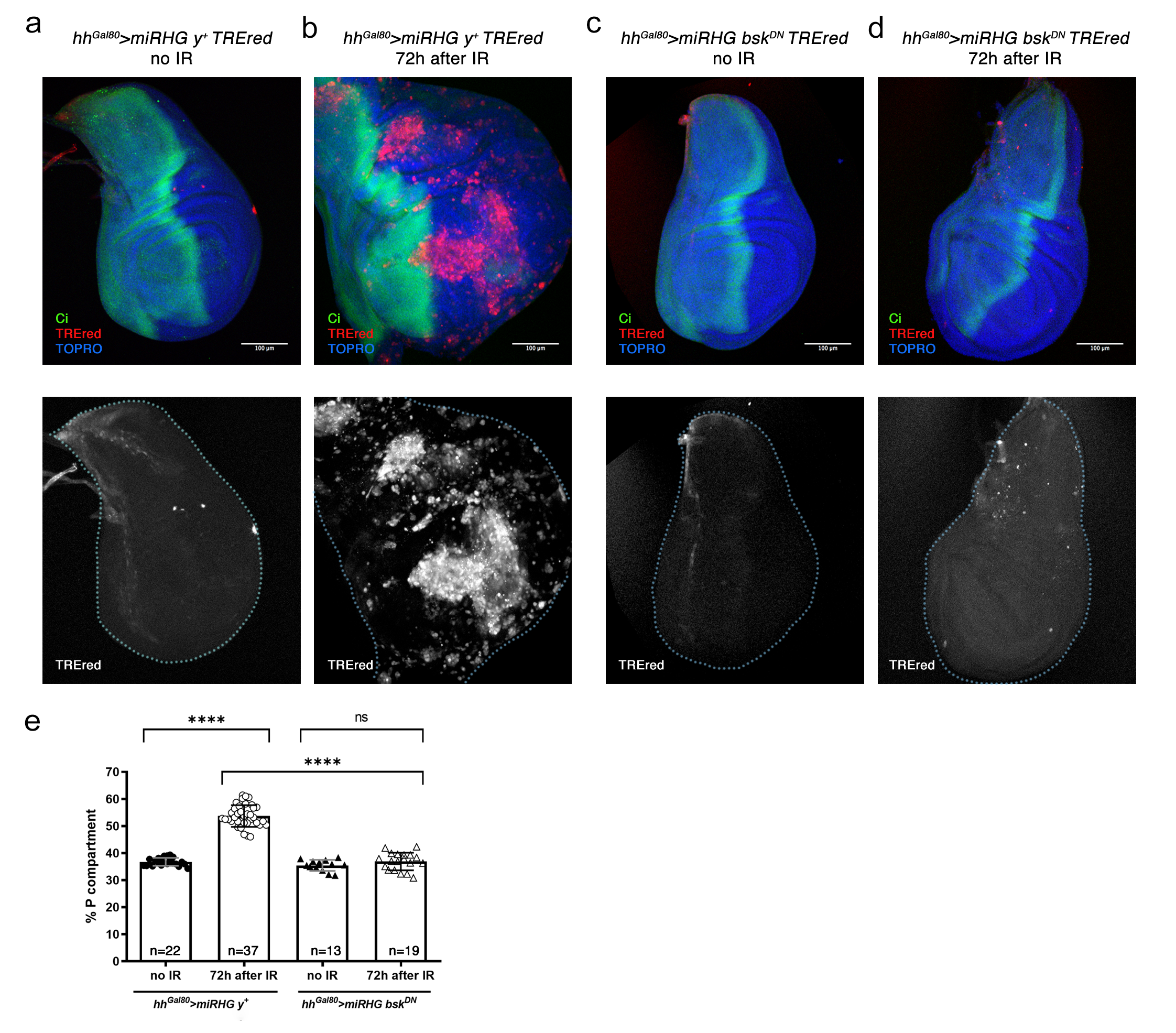

Supplement: Supplementary file 1 — Supplementary Figure 1 [file 41420_2023_1583_MOESM1_ESM.tif]

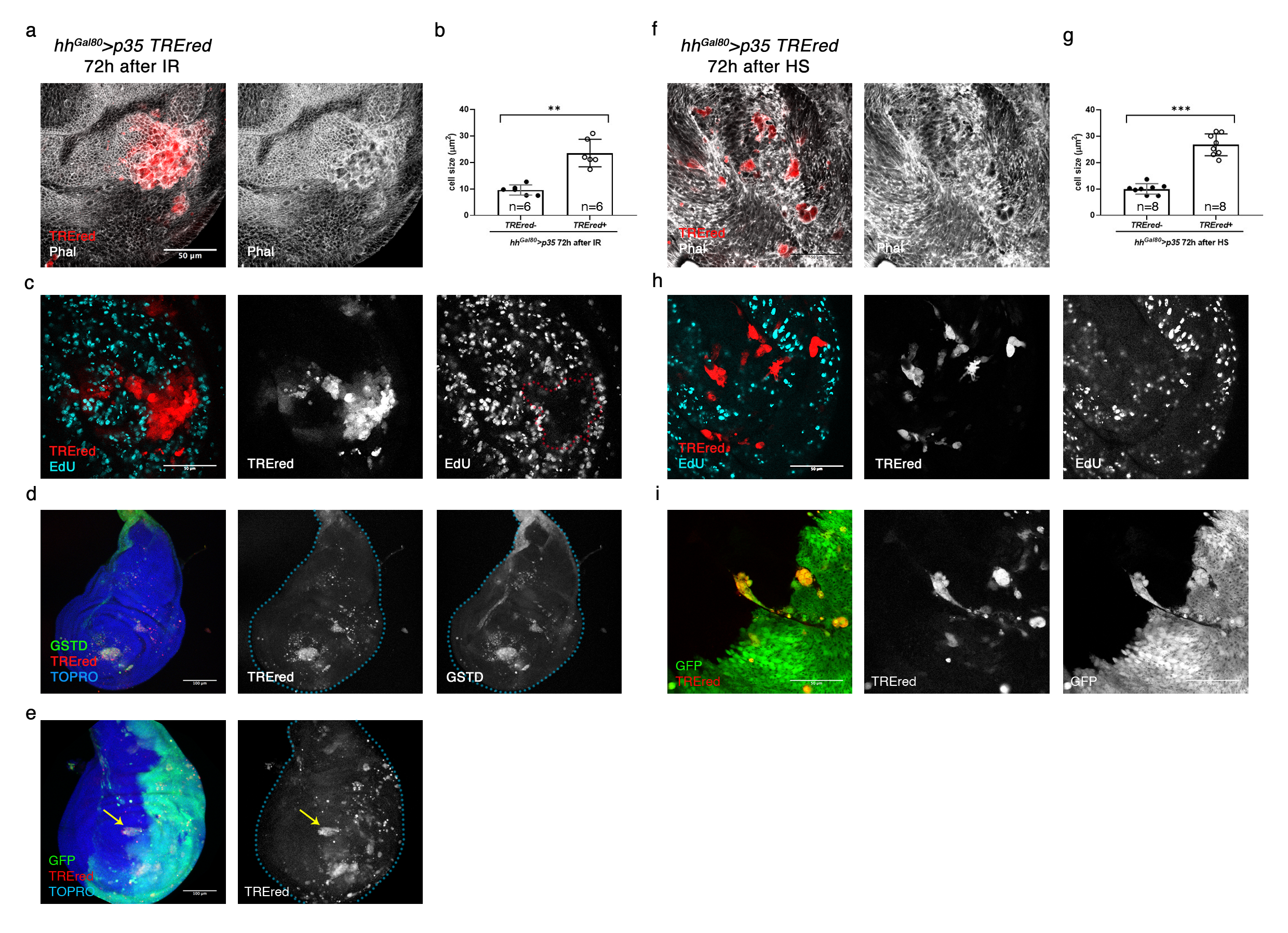

Supplement: Supplementary file 2 — Supplementary Figure 2 [file 41420_2023_1583_MOESM2_ESM.tif]

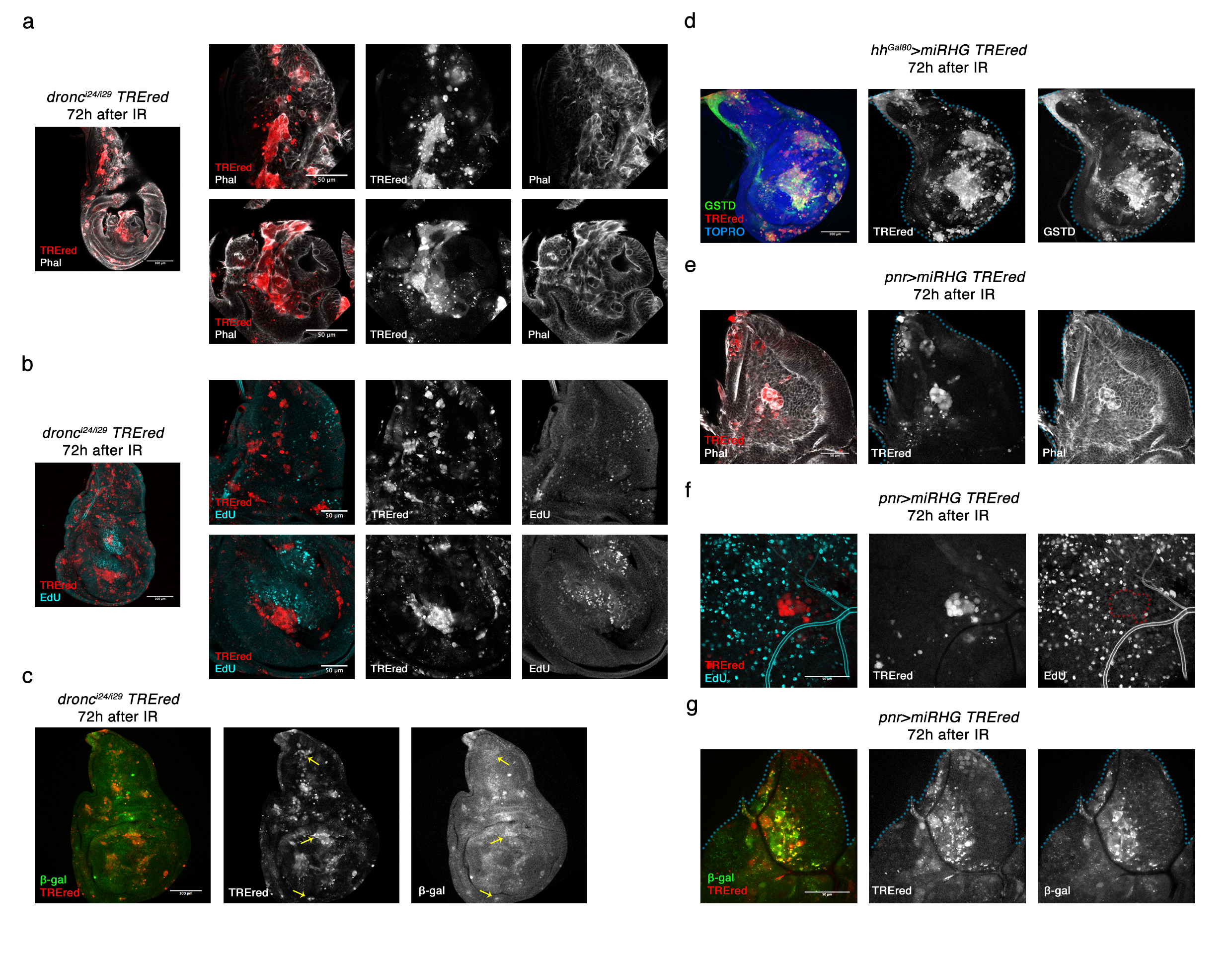

Supplement: Supplementary file 3 — Supplementary Figure 3 [file 41420_2023_1583_MOESM3_ESM.tif]
