# Supplementary material for: Lack of apoptosis leads to cellular senescence and tumorigenesis in Drosophila epithelial cells
Source: Cell Death Discov. 2023 Aug 2;9:281. doi: 10.1038/s41420-023-01583-y (PMC10397273; doi:10.1038/s41420-023-01583-y)
Supplement: Supplementary file 4 — Supplementary Figure legends [file 41420_2023_1583_MOESM4_ESM.docx]

**Supplementary** **Figure 1**

**Effect of irradiation (4000R) on apoptosis-deficient posterior compartment and role of JNK.**

Wing imaginal discs of the genotypes and treatments described above each image stained for TREred (red), TOPRO (blue) and Ci (green).

a) Non-irradiated disc of *hh^Gal80^>UAS-miRHG, TREred* phenotype shown normal size and virtually no cells expressing TREred.

b) Disc of the same genotype as in (a) 72h after IR. Note the significant increase of size of the posterior compartment, quantified in (e), and the accumulation of cells expressing the TREred construct.

c-d) The panels illustrate the response to IR of discs in which the posterior compartments cells contain a dominant negative form of the kinase Basket (*bsk^DN^*), which makes the JNK pathway ineffective. Note in (d) that the IR has no effect on discs of this genotype.

e) Quantification of the size of the posterior compartment in the genotypes and treatments indicated, represented as % of the posterior domain respect to the total area of the disc. n is indicated in the graph. Statistical analysis by one-way ANOVA when compared the mean of each column with the mean of the control as indicated. ****=p<0.0001 and ns=not significant.

**Supplementary** **Figure 2**

**The “undead” cells are senescent.**

a) Part of a wing disc of genotype *hh^Gal80^> p35, TREred* after 4000R IR. Phalloidin (white) staining reveals that the size of TREred cells is bigger than that of surrounding cells.

b) Graph that shows the quantification of the effect on cell size in the genotypes described (n=6 discs and each point is the average cell size of 10 cells). Statistically significant differences based on Student’s t test are indicated: **<p=0.01.

c) EdU incorporation (blue) shows that TREred cells do not divide, in contrast to surrounding cells.

d) Wing disc of genotype *hh^Gal80^>UAS-p35, TREred* after 4000R IR. TREred cells also show expression of the *GstD-lacZ* (green), a reporter of ROS production.

e) Invasion of the anterior compartment by TREred cells of posterior origin (yellow arrow).

f) TREred cells generated after 3h heat shock (HS) at 37ºCof the genotype *hh^Gal80^>UAS-p35, TREred* become bigger that surrounding cells, as revealed by Phalloidin staining (white).

g) Graph that shows the quantification of the effect on cell size in the genotypes described (n=8 discs and each point is the average cell size of 10 cells). Statistically significant differences based on Student’s t test are indicated: ***=p<0.001.

h) EdU incorporation (blue) shows that TREred cells do not divide, in contrast to surrounding cells in *hh^Gal80^>UAS-p35, TREred* after 72h of HS.

i) Invasion of the anterior compartment by TREred cells of posterior origin in *hh^Gal80^>UAS-p35, UAS-GFP, TREred* wing discs after 72h of HS.

**Supplementary** **Figure 3**

**Senescent features in *dronc^-^* discs and in the notum region.**

Irradiated discs mutant for the apical caspase Dronc staining with TREred and phalloidin (a), EdU (b) or β-galactosidase activity (c). Note that all TREred cells in the discs are bigger than surrounding cells (a), do not proliferate (b) and show high levels of β-galactosidase activity (c), both in the thoracic and the appendage region.

d) Irradiated disc of the genotype *hh^Gal80^>UAS-miRHG, TREred* showing that ROS production, visualized by the expression of the reporter gene *GstD*, takes place in all the TREred cells.

e) Phalloidin staining in irradiated disc of the genotype *pnr>UAS-miRHG, TREred* shows that TREred cells generated in the notum region are bigger than surrounding cells.

f) EdU incorporation (blue) shows that TREred cells do not divide, in contrast to surrounding cells.

g) TREred cells in the notum region produces high levels of β-galactosidase activity assay.
